# Small bowel adenocarcinoma of the jejunum: a case report and literature review

**DOI:** 10.1186/s12957-016-0932-3

**Published:** 2016-07-04

**Authors:** Jie Li, Zhiliang Wang, Na Liu, Junfeng Hao, Xin Xu

**Affiliations:** Department of General Surgery, The Second Affiliated Hospital, Xi’an Jiaotong University Health Science Center, Xi’an, 710004 China; Department of Cell Biology and Genetics, School of Basic Medical Sciences, Xi’an Jiaotong University Health Science Center, Xi’an, 710061 China

**Keywords:** Small bowel adenocarcinoma, Rarity, Diagnosis, Surgery, FOLFOX

## Abstract

**Background:**

In practice, small bowel cancer is a rare entity. The most common histologic subtype is adenocarcinoma. Adenocarcinoma of the small bowel (SBA) is challenging to diagnose, often presents at a late stage and has a poor prognosis. The treatment of early-stage SBA is surgical resection. No standard protocol has been established for unresectable or metastatic disease.

**Case presentation:**

We report here on a 26-year-old man with SBA in the jejunum, lacking specific symptoms and with a delay of 6 months in diagnosis. The diagnosis was finally achieved with a combination of balloon-assisted enteroscopy, computed tomography scans, positron emission computed tomography scans and the values of carcino-embryonic antigen and carbohydrate antigen 19-9. The patient underwent segmental intestine with lymph node resection, followed by eight cycles of FOLFOX palliative chemotherapy with good tolerance. As of the 11-month postoperative follow-up, there has been no evidence of recurrent disease.

**Conclusions:**

This case is reported to arouse a clinical suspicion of SBA in patients with abdominal pain of unknown cause. We also provided evidence in this case of a response to palliative chemotherapy with FOLFOX. Because the incidence of SBA is very low, there is a need for further studies to evaluate the possible application of newer investigative agents and strategies to obtain a better outcome within the framework of international collaborations.

## Background

Small bowel cancer is a rare malignancy that comprises less than 5 % of all gastrointestinal malignancies. The estimated annual incidence is 0.3–2.0 cases per 100,000 persons, with a higher prevalence rates in the black population than the white, and has been recently increasing [1, 2]. It is most frequently diagnosed among people aged 55–64, with the incidence increasing after age 40. The current 5-year survival rate in the USA is 65.5 %; cancer stage at diagnosis has a strong influence on the length of survival [3].

Small bowel cancer has four common histological types: adenocarcinoma (30–40 %), carcinoid tumour (35–42 %), lymphoma (15–20 %), and sarcoma (10–15 %) [4]. Adenocarcinoma of the small bowel (SBA) is most commonly located in the duodenum (57 %), while 29 % of cases are located in the jejunum and 10 % in the ileum [5]. Clinical presentation of SBA is nonspecific abdominal discomfort, such as abdominal pain, nausea, vomiting, gastrointestinal bleeding and intestinal obstruction, which leads to an average delay of 6–10 months in diagnosis [6]. Due to the rarity of this cancer, there have been no good screening methods developed for SBA; little is known about the clinical characteristics, treatment modalities or prognosis of patients with SBA, especially in Asians.

Here, we report on a 26-year-old man with SBA in the jejunum, without specific symptoms. He was diagnosed until he had an incomplete small bowel obstruction, with a delay of 6 months in diagnosis. Segmental intestine with lymph node resection was performed, followed by eight cycles of FOLFOX palliative chemotherapy. The patient was doing well as of his last follow-up.

## Case presentation

The patient was male, 26 years old and had no specific underlying or family disease. Six months ago, he experienced an episodic attack of distending pain in his left lower quadrant, nausea and vomiting; he was treated with oral drugs at a local hospital. However, his symptoms were not completely relieved, and were later aggravated. A normal abdominal X-ray suggested incomplete small bowel obstruction. He was admitted to our hospital.

**Fig. 1:**
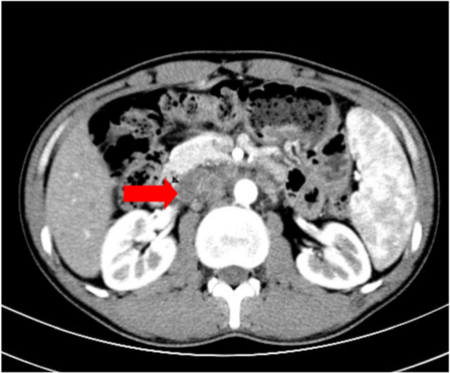
CT scan image (axial view) showing many swollen lymph nodes adjacent to the abdominal aorta in the retroperitoneal space but no discernible mass

**Fig. 2:**
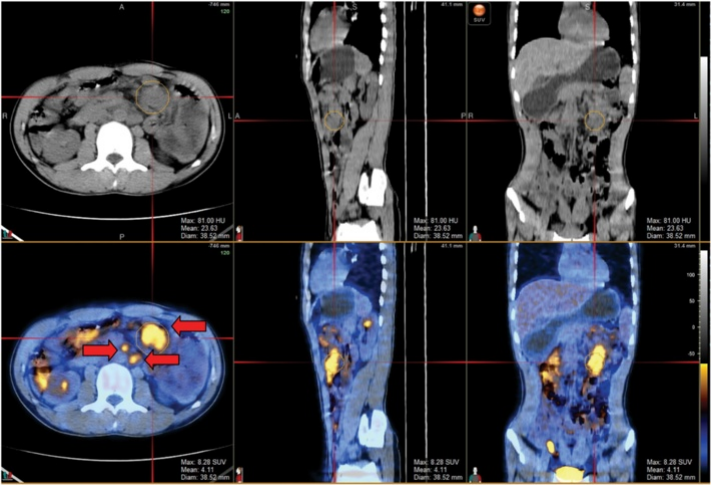
PET/CT scan image showing abnormal accumulations of ^18^F-FDP in many intestinal segments and also in many retroperitoneal swollen lymph nodes, indicating hypermetabolism disease, with a high possibility of a malignant disease

**Fig. 3:**
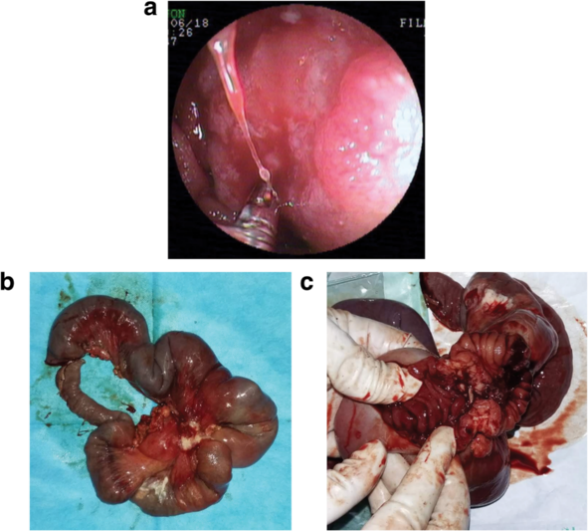
**a** DBE examination showing at the upper jejunum; lumen was narrowed by an irregular protrusive tumour. **b** About 40 cm of the involved jejunum, mesentery and vessels were resected. **c** Tumour involved the entire wall of the small intestine

The patient visited our hospital without any complaints. Physical examination revealed a soft abdomen with tenderness in the left lower quadrant. No mass was palpated in the abdomen. When his abdominal pain occurred, a peristaltic wave could be observed around the navel. Laboratory tests showed no anaemia or leukocytosis. Examination of tumour-associated antigens showed a prominent high levels of carcino-embryonic antigen (CEA) at 29.17 ng/ml and carbohydrate antigen 19-9 (CA 19-9) at 970.3 U/ml. Abdominal computed tomography (CT) scans showed many swollen lymph nodes adjacent to the abdominal aorta in the retroperitoneal space (Fig. 1) but no discernible mass. Positron emission computed tomography (PET)/CT scans revealed abnormal accumulations of ^18^F-FDP in many stiffening intestinal segments and also in many retroperitoneal swollen lymph nodes, indicating hypermetabolism disease, with a high possibility of a malignant disease (Fig. 2). Gastroscopy and enteroscopy showed that the stomach, colon and rectum were normal. However, double-balloon enteroscopy (DBE) and the following biopsy revealed at the upper jejunum that most of the lumen was obstructed by an irregular protrusive tumour of gastrointestinal origin (Fig. 3a).

**Fig. 4:**
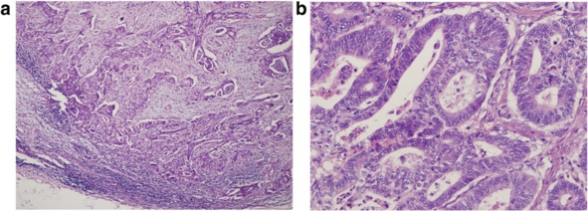
Microscopic images of the tumour from the pathologic specimen; haematoxylin and eosin staining. **a** Low-power magnification (×100) showing a moderately differentiated adenocarcinoma of the jejunum with invasion into the lymph nodes. **b** High-power magnification (×400) showing adenocarcinoma

**Fig. 5:**
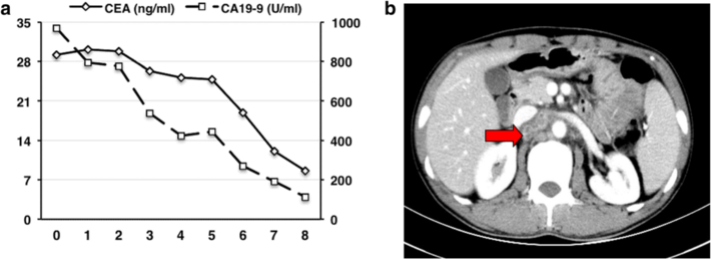
**a** Values of CEA (*left Y-axis*) and CA 19-9 (*right Y-axis*) decreased gradually as the eight cycles of FOLFOX palliative chemotherapy progressed. **b** CT scan image (axial view) showing swollen lymph nodes adjacent to the abdominal aorta in the retroperitoneal space lessened after chemotherapy

Because of the symptoms of intestinal obstructions and a high possibility of advanced stage, the patient underwent segmental resection of the jejunum. At laparotomy, a 5 × 5 cm round mass with no distinct boundary was present at the jejunum (25 cm from the ligament of Treitz). The mass involved the entire wall of the small intestine and directly invaded the neighbouring mesentery. There were many enlarged lymph nodes around the superior mesenteric vein and the first and second jejunal arteries in the involved mesentery. There was no evidence of metastatic lesions in the peritoneum or liver during intraoperative inspection of all quadrants of the abdominal cavity. We performed a radical resection with 40 cm of the jejunum and the involved mesentery, vessels and lymph nodes (Fig. 3b, c). Pathologic examination revealed a moderately differentiated adenocarcinoma with metastasis to seven out of 14 resected lymph nodes (Fig. 4); free surgical margins were achieved. The tumour was staged as T4N2M0, stage IIIB disease [7]. Genetic studies of the specimen revealed that it had low expression of thymidylate synthase (TS) and excision repair cross-complementing gene 1 (ERCC1), sensitive to fluoropyrimidine and platinum [8]. He was started on palliative chemotherapy with FOLFOX for a total of eight cycles. He tolerated chemotherapy well, and the values of CEA and CA 19-9 decreased gradually as the chemotherapy progressed (Fig. 5a). CT scans also showed that the swollen lymph nodes adjacent to the abdominal aorta were significantly lessened (Fig. 5b). As of the 11-month postoperative follow-up, there has been no evidence of recurrent disease.

### Discussion

Whereas the small bowel represents 75 % of the length of the digestive tract and 90 % of the absorptive mucosal surface area, tumour of the small bowel is rarer than other gastrointestinal malignancies. The possible explanations include the high levels of IgA and the more rapid transit in the small bowel compared to the large bowel. Little bacteria and more sensitivity to stress in the small bowel also contribute to the low tumour incidence [9]. Though small bowel cancer normally occurs in elderly patients [3], in this case, it was found in a 26-year-old young man. The mass remained undetectable until he had an incomplete small bowel obstruction with lymph node metastasis. This was similar with studies, in which diagnosis of SBA was mainly obtained at advanced stages; ~40 % of patients have lymph node metastasis (stage III), and 35 to 40 % have distant metastasis (stage IV) [6, 10].

The symptoms of SBA are initially nonspecific abdominal discomfort; diagnosis is delayed and usually in the context of emergency involving an occlusion (40 %) or bleeding (24 %) [6], which is similar to the presentation of our patient. For diagnosis of SBA, CT scans have an overall accuracy rate of 47 % [11]. While CT scans can detect the lesions, they cannot provide precise data of the intestinal mucosa and miss some small or flat lesions. The PET/CT technique is being used to differentiate small intestinal malignant tumours from benign ones. The uptake of ^18^F-FDG is related to tumour size, infiltration and lymph node metastasis; the higher the uptake of ^18^F-FDG, the higher the tumour invasiveness [12]. Gastroscopy and enteroscopy can be appropriate if the tumour is located close to the proximal duodenum or far from the terminal ileum. The rest of the small bowel cannot be accessed without the use of video capsule endoscopy (CE) or DBE. The definite diagnostic yield of CE is only 20–30 %, while DBE accounts for 60–70 % of the diagnostic yield for intestinal diseases [13]. However, CE is suitable for diagnosing scattered, small and multiple lesions, as well as active bleeding; it is convenient, non-invasive, secure and comfortable. In contrast, the DBE procedure is uncomfortable, less tolerated and difficult to complete; these factors influence its diagnosis [13]. A baseline plasmatic CEA and CA 19-9 assay is necessary, especially in cases of advanced disease because CEA and CA 19-9 levels are of prognostic value [14]. In this case, the diagnosis was achieved by the combination of the DBE results, CT images, PET/CT images and the values of CEA and CA 19-9.

Surgical resection with clear margins and regional lymph node resection remains the treatment of choice in localized SBA; indeed, it is often required even in metastatic SBA due to the high probability of obstruction or severe haemorrhage [15]. To date, there has been no standard chemotherapy regimen against SBA. Several studies have explored the role of palliative chemotherapy in advanced SBA. Hong et al. [16] have shown in stage IV patients who received palliative chemotherapy that overall survival (OS) increased significantly compared to those who did not receive chemotherapy (8 vs. 3 months, *p* = 0.025). Ecker et al. [17] have shown that median OS was superior for patients with resected stage III SBA who were receiving chemotherapy versus those who were not (42.4 vs 26.1 months, *p* < 0.001). As for the Asian population, Mizyshima et al. [18] showed that, in patients with non-curative resection or unresectable distant metastasis, the response rate to chemotherapy was 31.6 %, and the 3-year OS rate was significantly higher compared to the response rate without chemotherapy (26.3 vs. 13.8 %; *p* = 0.008). Several chemotherapy drugs have also been evaluated in the treatment of metastatic SBA. Zaanan et al. [14] have shown that the median OS in advanced SBA patients treated with FOLFOX was 17.8 months, the longest survival among different chemotherapy regimens. Two phase II studies have been conducted to evaluate the efficacy of different chemotherapy regimens in advanced SBA: the response rates were around 50 %, the median progression-free survivals 7.8 and 11.3 months and the median OS 15.2 and 20.4 months [19, 20]. Newer agents, such as endothelial growth receptor (EGFR) antibody drugs, and newer combinations are being explored as the second line for improved treatment of advanced SBA [21]. From limited clinical reports, a combination of fluoropyrimidine with platinum compounds (FOLFOX or CAPOX) has been proposed as the first-line treatment for palliative chemotherapy in metastatic SBA treatment [22]. In view of the results of genetic studies, the patient underwent palliative chemotherapy for eight cycles of FOLFOX and was doing well as of his last follow-up.

## Conclusions

We report a rare case of jejunum adenocarcinoma in a young man in China. Diagnosis of SBA remains a challenge. A physician’s suspicion and awareness is crucial in patients with abdominal pain of unknown cause. Patients with delayed diagnosis often have a poorer prognosis. Surgery remains the primary treatment. In this case, we noticed a response to palliative chemotherapy with FOLFOX. Because the incidence of SBA is very low, there is a need for further studies to evaluate the possible application of newer investigative agents and strategies to obtain a better outcome within the framework of international collaborations.

## Abbreviations

CA 19-9, carbohydrate antigen 19-9; CEA, carcino-embryonic antigen; CT, computed tomography; DBE, double-balloon enteroscopy; ERCC1, excision repair cross-complementing gene 1; PET, positron emission tomography; SBA, adenocarcinoma of the small bowel; TS, thymidylate synthase
